# Telehealth to Expand Community Health Nurse Education in Rural Guatemala: A Pilot Feasibility and Acceptability Evaluation

**DOI:** 10.3389/fpubh.2017.00060

**Published:** 2017-03-29

**Authors:** Kelly A. McConnell, Lyndsay K. Krisher, Maureen Lenssen, Maya Bunik, Saskia Bunge Montes, Gretchen J. Domek

**Affiliations:** ^1^Center for Global Health, Colorado School of Public Health, Aurora, CO, USA; ^2^Department of Pediatrics, University of Colorado Anschutz Medical Campus, Aurora, CO, USA; ^3^Center for Health, Work & Environment, Colorado School of Public Health, Aurora, CO, USA; ^4^Center for Human Development at the Southwest Trifinio, Retalhuleu, Guatemala

**Keywords:** community health nursing, child health, telehealth, education, medical, rural health services, Guatemala

## Abstract

Telehealth education has the potential to serve as an important, low-cost method of expanding healthcare worker education and support, especially in rural settings of low- and middle-income countries. We describe an innovative educational strategy to strengthen a long-term health professional capacity building partnership between Guatemalan and US-based partners. In this pilot evaluation, community health nurses in rural Guatemala received customized, interactive education *via* telehealth from faculty at the supporting US-based institution. Program evaluation of this 10 lecture series demonstrated high levels of satisfaction among learners and instructors as well as knowledge gain by learners. An average of 5.5 learners and 2 instructors attended the 10 lectures and completed surveys using a Likert scale to rate statements regarding lecture content, technology, and personal connection. Positive statements about lecture content and the applicability to daily work had 98% or greater agreement as did statements regarding ease of technology and convenience. The learners agreed with feeling connected to the instructors 100% of the time, while instructors had 86.4% agreement with connection related statements. Instructors, joining at their respective work locations, rated convenience statements at 100% agreement. This evaluation also demonstrated effectiveness with an average 10.7% increase in pre- to posttest knowledge scores by learners. As the global health community considers efficiency in time, money, and our environment, telehealth education is a critical method to consider and develop for health worker education. Our pilot program evaluation shows that telehealth may be an effective method of delivering education to frontline health workers in rural Guatemala. While larger studies are needed to quantify the duration and benefits of specific knowledge gains and to perform a cost-effectiveness analysis of the program, our initial pilot results are encouraging and show that a telehealth program between a US-based university and a rural community health program in a low- and middle-income country is both feasible and acceptable.

## Introduction

Over one billion people globally have limited access to health services, with many living in rural areas in low- and middle-income countries (LMICs) (1). One important way to reduce this growing burden is to increase training of frontline health workers worldwide. In particular, community health nurses (CHNs) are vital health-care workers who can extend healthcare in rural areas, especially in LMICs. These nurses are trained health workers based in central health centers who meet the needs of local people by visiting and providing care at individual homes and community group visits. The importance of community health workers and CHNs has been widely recognized on an international scale. In 2008, the World Health Organization and the Global Workforce Alliance challenged the international community “to make sure that everyone has access to a suitable, trained and motivated health worker as part of a functioning health system” (1). In order to ensure the ongoing effectiveness of CHNs, organizations must provide continuing education to health workers.

Telehealth is defined as “the use of electronic information and telecommunications technologies to support long distance healthcare, patient and professional health-related education, public health and health administration” (2). This technology has proven to be a promising method of providing continuing education for healthcare providers (3) as well as supporting clinical services for patients (4). Multiple studies demonstrate effective distance education to rural locations in the United States using telehealth (5–7). However, there remains limited evidence of the cost-effectiveness and the acceptability by both patients and health-care providers of using this technology, and most research to date has been conducted within higher income countries. Recent studies have begun to show promise in addressing the disparity of medical specialists in LMICs by expanding access to sub-specialty training through telehealth (8, 9). Several organizations link multiple groups across geographical and cultural boundaries to provide educational videoconferencing, including the Reseau en Afrique Francophone pour la Telemedicine (10–12), the KwaZulu-Natal Experience (13), and the Global Educational Toxicology Uniting Project (14). Additionally, several programs have explored collaborations between academic institutions/hospitals in higher income countries and LMICs to provide medical education *via* teleconference, including in specialties such as obstetrics and gynecology (15), emergency and trauma care (16), anesthesia (17), and surgical skills training (18). While much of the rapidly expanding research for Internet-based remote education in LMICs is focused on the continuing medical education of physicians and trainees, a study in Malaysia showed that nurses and their remote lecturers responded positively to e-learning, noting that communication and interaction are key components to a teleconferencing format of teaching (19). Furthermore, during the recent Ebola epidemic, a tablet computer tutorial application was successfully used to train frontline health workers by improving knowledge and attitudes surrounding the virus (20). While telehealth technology holds great promise in educating health professionals in LMICs, very few studies have assessed the feasibility and acceptability of this teaching method with frontline health workers such as CHNs.

## Background and Rationale

Our organization, the Center for Global Health at the Colorado School of Public Health, has partnered with a local agricultural company, AgroAmerica, in the coastal lowlands of southwest Guatemala to create a health center and community health program that improve general health and access to healthcare to several small communities that make up a rural population of approximately 30,000 people (21). This area in Guatemala is cultivated with crops for export, primarily bananas and palm oil, owned by large agro-business enterprises, and the rural population struggles with poverty and lack of access to health, education, and reliable clean water. Our partnership is a unique relationship between a university, a private business, and a community. The program is largely funded by the Guatemalan agricultural company with our university establishing and supporting medical services through a Guatemalan-staffed clinic, laboratory, and pharmacy as well as community-based health programs for maternal and early childhood health. The CHNs in our program conduct home and group visits for pregnant women and children up to 3 years of age. They follow women throughout pregnancy, monitoring expectant mothers and providing interventions that improve prenatal care and delivery. Once the baby is born, the mother and child transition to an early childhood health and development segment of the program that combines a series of neonatal home visits, community education sessions, and mother–child interactive group visits to enhance the health and development of children from birth to 3 years of age. CHNs travel throughout the communities performing assessments of general health, child development and anthropometrics, and providing anticipatory guidance and basic health advice.

In the summers of 2014 and 2015, a general lack of lactation knowledge and support was identified within our program. As a result, team members from our institution provided in-person breastfeeding training to the CHNs while present in Guatemala as baseline education. Following the 2015 program, both the CHNs and the instructors wanted to continue the breastfeeding educational program. However, as in many rural health-center locations in LMICs, a great deal of time and resources are needed to send instructors to the local site for in-person trainings. As many rural health centers reach communities outside of main cities and common thoroughfares, travel to these sites frequently involves extended time and costs, limiting teaching faculty to a few longer trips and limiting teaching to short periods of intense training. From our organization, it costs approximately 3000 USD to send one faculty member to Guatemala for 2 weeks, which includes airfare, room and board, and local transportation but does not even include faculty salary, which varies considerably. In the initial years of program development and implementation, our institution would spend around 50,000 USD annually to send faculty members to our rural site, which quickly became a financial limitation. For programs using local faculty, instructors can provide sustained regular contact with CHNs allowing for interactive discussions over time, including case reviews related to the teaching. For distance programs and partnerships such as ours, however, regular training of CHNs creates the need for a more innovative and cost-effective education delivery method using information and communication technology such as telehealth.

For this reason, continuation of the initial breastfeeding curriculum was initiated *via* a videoconferencing software program at our institution. Developed in response to the expressed needs of the CHNs, topics then expanded to more generalized child health topics in rotation with breastfeeding lectures. The initial pilot period of July–November 2015 demonstrated general feasibility and satisfaction among team members and CHNs, and the team proceeded with a planned curriculum that included knowledge assessments and formal evaluations. The current program evaluation for this telehealth curriculum aimed to prove knowledge gains in child health topics, assess satisfaction and convenience with telehealth technology, demonstrate connection between learners and instructors, and identify challenges in delivery.

## Methods

**Figure 1:**
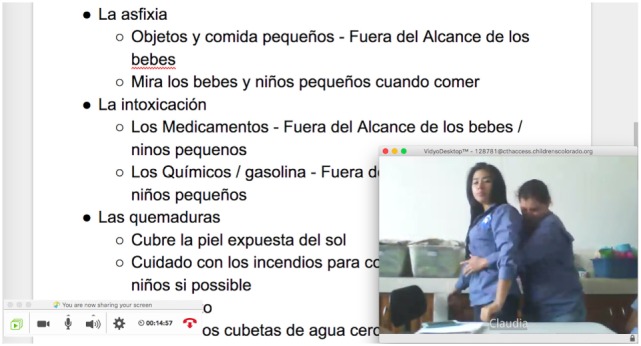
**Example of lead instructor’s Vidyo^©^ screen**.

This pilot was considered to be program evaluation rather than human subject research by the Colorado Multiple Institutional Review Board, and therefore, informed consent was not required. The CHNs were aware of the evaluation process during the program. The program evaluation took place between February and May 2016. Child health lectures were organized into two blocks of five topics each and were selected based on CHN preferences and instructor ability. Lectures covered the following topics: anemia, ear infections, zinc, urinary tract infections, antibiotics, vaccines, obesity, vitamin A, injury prevention, and burns. We used the videoconferencing software program Vidyo^©^ (22), licensed by the Telehealth Department at our institution and available free of charge to our program. Vidyo^©^ is a high-definition videoconferencing platform with an encrypted signal between several computers or mobile devices. The system has smoothing capabilities due to an iterative signal to allow for more natural communication. The platform runs on our institution’s secure network. One of the team members acted as lead instructor by using the Vidyo^©^ platform and sharing the screen with supplementary documents or slides on the topic. Other team members joined lectures as assistant instructors or observers. The lectures consisted of approximately 30 min of didactic teaching with an additional 15 min for questions, case presentations, and discussions of current cases and experiences in the community, providing a total time of approximately 45 min connected *via* telehealth. Learners viewed the shared supplementary teaching materials and the instructors simultaneously on their screen. The lead instructor viewed the learners as well as the instructor’s home screen with supplementary teaching materials (Figure 1). Lectures were given in Spanish, the native language of the learners, but there was intermittent discussion among instructors in English for clarification. Learners were able to access the teaching documents after the lecture. The educational sessions were not recorded due to the need to maximize bandwidth availability at the Guatemala location.

Internet connection for the instructors was home or office Wi-Fi or cellular network on their personal or work computers, tablets, or phones. The devices connected securely to our institution’s network through Vidyo^©^. The CHNs used an established Wi-Fi connection at the clinic campus in Guatemala freely available to employees. The Internet at the clinic costs 5,300 Guatemalan Quetzales or approximately 700 USD per month for connection speeds of 6 mbps, which is paid by the Guatemalan agricultural company. This Internet connection is used for maintaining electronic medical records at the clinic and data downloads for multiple ongoing research studies. There were no additional costs to our program for the use of the clinic Internet. No evaluation of specific bandwidth usage was done for this assessment.

### Evaluation Instruments

Evaluation of the program consisted of pre- and post-knowledge assessments by the learners as well as quality and satisfaction evaluations by both learners and instructors. All testing and evaluations were done through Google Forms^©^ (Google Docs, RRID:SCR_005886). The anonymous survey data were collected and stored in Google Docs and then further processed in Microsoft Excel^©^. Two blocks of five lectures were given. Each block was preceded by a pre-test, consisted of one lecture per week for 5 weeks and was followed by a posttest after the last lecture was given. Pre- and posttests assessed knowledge of each of the five topics per block, with 20 points available per topic and an overall 100 points per test for each of the two blocks. Each lecture was followed by a quality and satisfaction evaluation assessing content, technology, and connection between instructors and learners on a 4-point Likert scale. Instructors completed a separate evaluation assessing technology, convenience, and connection. Both evaluations requested details on technology difficulties and general feedback on the teaching. As technical problems can affect the students’ perceptions of quality (23), technical quality (e.g., audio, video, and time to connect) was measured with each lecture. All surveys and tests were completed anonymously to allow for candid responses.

### Analysis

Survey responses from instructors and learners were combined across 10 lectures for mean Likert scores (1–4 range) with population SD. Percent of “agree” responses (scores 3 and 4) was also calculated per survey question. Survey questions were grouped into assessments of lecture content, technology, and connection among instructors and learners. Knowledge gain was measured by percent improvement per subject and overall pre- to posttest scores. Due to the small number of students, instructors, and lectures, correlation among survey responses per lecture was not performed.

## Results

### Demographics

The seven CHN learners in this program had completed either auxiliary nursing school (*n* = 4, 1 year post high school) or professional nursing school (*n* = 3, 3 years post high school). All CHNs were female, averaging 25 years of age and 5 years post-completion of nursing school. The lead instructor was a pediatrician at our institution (Kelly A. McConnell). Additional instructors were pediatricians (Maya Bunik and Gretchen J. Domek), a pediatric nurse practitioner (Maureen Lenssen), and a recent medical school graduate from Guatemala (Saskia Bunge Montes). All instructors had been to the site in rural Guatemala and had been involved with the implementation of the community health program.

### Knowledge Gain

**Figure 2:**
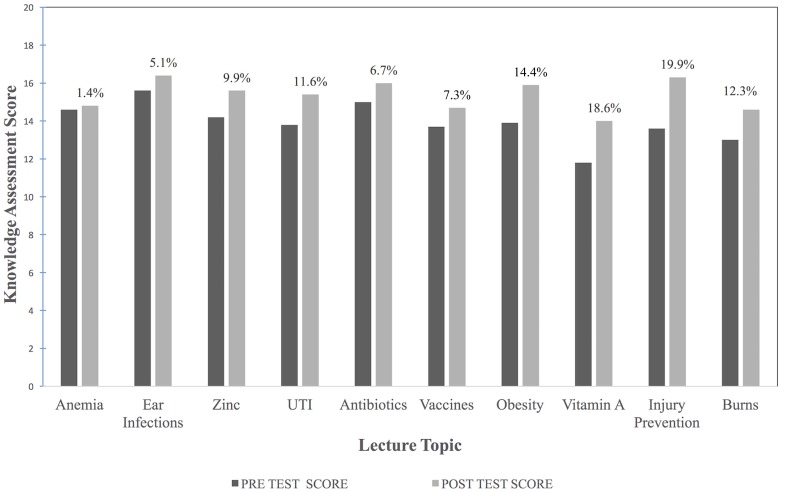
**Pre- to posttest knowledge gain and percent improvement**.

The percent increase of the mean for each lecture ranged from 1.4 to 19.9% (Figure 2) with an overall average increase of 10.7% among all topics. Overall, the correct score for each lecture improved from a mean of 13.9 to 15.4 out of 20 possible points. Test responses were not paired or adjusted for non-attendance of certain lectures as surveys and tests remained anonymous.

### Learners’ Evaluation

There was an average of 5.5 learners present per lecture. Responses from the learners were overall positive, especially regarding lecture content with 98% or greater agreement with each positive statement (Table 1). The technology questions regarding ease of use and convenience were also strongly positive with at least 98% agreement. The ability to hear the instructor (94.5% agreement) was greater than the ability to see the instructor (87.3% agreement), likely due to greater signal strength required for video compared to audio delivery. Statements related to the connection to the instructor were agreed upon 100% of the time except when asked if the lecture *via* telehealth was as good as in person (94.5% agreement). The amount of time to connect to the system was most frequently 5–10 min but took up to 15 min. Three lectures were rescheduled due to a lack of Internet connection at the site in Guatemala; two were done later in the same day and one was rescheduled to a different day. Overall, the CHNs were extremely satisfied with the lecture delivery *via* telehealth as well as the direct teaching.

**Table 1:** **Learner and instructor evaluation responses**.

	Mean Likert score^a^	SD (population)	Number of “disagree” responses (Likert score 1 or 2)	Number of “agree” responses (Likert score 3 or 4)	Percent with an “agree” response
**Learners: lecture content**					
I learned the stated objectives	3.76	0.43	0	55	100
This topic and content are useful to my daily work	3.83	0.36	0	55	100
My work in the community is going to change/improve through this lecture	3.75	0.48	1	54	98.2
This lecture improves my knowledge of this topic	3.83	0.37	0	55	100
The quantity of the information included in this teaching was appropriate	3.74	0.44	0	55	100
The quantity of material in this lecture is appropriate for teaching *via* telehealth	3.67	0.47	0	55	100
**Learners: technology**					
I could hear the instructor well	3.67	0.58	3	52	94.5
I could see the instructor well	3.43	0.76	7	48	87.3
The system is easy to use	3.67	0.51	1	54	98.2
This teaching was convenient in my schedule of daily work	3.72	0.45	0	55	100
**Learners: connection to instructor**					
This mode of teaching maintained my interest	3.74	0.44	0	55	100
I feel connected to the instructor	3.70	0.46	0	55	100
I believe the instructor cared about my learning	3.83	0.37	0	55	100
This lecture was as good *via* telehealth as in person	3.46	0.60	3	52	94.5
**Instructors: technology**					
I could see the learners well	2.86	1.01	9	13	59.1
I could hear the learners well	3.36	0.71	3	19	86.4
The system is easy to use	3.73	0.45	0	22	100
This format was convenient for me	3.95	0.21	0	22	100
**Instructors: connection to the learners**					
I feel connected to the learners	3.09	0.60	3	19	86.4

*^a^Answered on a 4-point Likert scale: 1 = strongly disagree, 2 = disagree, 3 = agree, 4 = strongly agree*.

### Instructors’ Evaluation

An average of two instructors was present for each lecture, one lead instructor and generally one additional team member. Audio quality was again reported to be better than video quality with “I could see the learners well” agreed upon only 59.1% of responses compared to 86.4% for audio (Table 1). Ease and convenience statements were 100% agreed upon, reflective of the system in which instructors joined from their personal or work computers or phones at their convenience if available. The amount of time to connect was nearly immediate in all reports from the instructors except two instances in which an observer was not able to connect. Feelings of connection to the learners by the instructors were lower at 86.4% than was reported from learners who reported 100% agreement.

## Discussion

Our pilot evaluation contributes important results on the feasibility and acceptability of using telehealth technology to train CHNs in a rural LMIC, an area where very little research currently exists. In fact, we are not aware of another study using telehealth to train CHNs in a LMIC *via* an academic partnership. Our study shows that with an overall improvement of more than 10% in pre- to posttest knowledge scores, teaching CHNs through telehealth was effective for knowledge gain specific to the 10 topics taught. CHNs also reported that these teachings were useful and would be impactful for their daily work in the community. Some lectures had a greater improvement than others, which may be related to the difficulty of the test questions, baseline knowledge, quality of the technology, and lecture content. Knowledge gain did not seem to be influenced by the amount of time between each lecture and the final posttest.

High rates of satisfaction and convenience with the telehealth system were demonstrated in our surveys from both learners and instructors. This is a critical component of feasibility and program evaluation, enabling continued investment from both parties. As telehealth involves remote interaction, connection is both difficult and important to maintain. Our instructors are culturally much more experienced with in-person teaching, while the CHNs have less experience with typical classroom didactic education, possibly reducing expectations. This may explain the higher satisfaction from the CHNs compared to the instructors. Overall, a sense of personal connection in regards to the teaching was felt by both parties, and importantly, the CHNs felt that the instructors cared about their learning. We think that there is likely a correlation between video quality and feelings of connection, but our dataset is not large enough for this analysis.

### Strengths

A major strength of our study was the low start-up costs and minimal resources needed to develop and implement a telehealth program. We used existing institutional computer equipment, Internet connections, teleconferencing software, and office space, including a well-established videoconferencing system, Vidyo^©^ (22), as well as Internet access readily available to each participant. This substantially reduced the costs of initiation. While Vidyo^©^ is provided free-of-charge at our institution, other videoconferencing software programs, such as Skype, have basic software packages that are easily accessible, free, and have been used by other e-learning programs (15, 17). Our team also had access and support from the Telehealth Department at our institution, providing infrastructure, technical expertise, and connection troubleshooting, all of which are critical elements to any e-learning programs. In general, Internet connectivity was strong during our study with few failed connections or technical difficulties experienced. Additionally, our educational sessions were all scheduled during protected academic time for our faculty and regular working hours for our CHNs, adding no further salary costs.

Another major strength to our program was the interactive, repetitive, and case-based learning style that we incorporated into each educational session. Research has shown the importance of active-learning exercises, personal interactions and feedback, intensive practice and repetition, and peer discussion in improving learning outcomes (24–26). A major advantage to using telehealth technology over web-based e-learning is the ability to have real-time interaction and active participation that facilitates asking questions, receiving clarifications, and discussing case presentations. In a review of e-learning in LMICs (27), lack of face-to-face interaction is discussed as a challenge to educational effectiveness. Additionally, all members of our team have traveled to the site in Guatemala, met the CHNs in-person, and been involved in multiple elements of the project, allowing a stronger connection, we believe, compared to telehealth contact alone. This participation with and knowledge of the community health program also allowed the team to create content directly applicable to the situations in which the CHNs worked daily.

While not assessed in our small pilot evaluation, there are likely to be significant economic, environmental, and personal benefits to such a program. We believe that increased education will improve the work of the nurses within the community since the lectures were targeted to improve CHN identification of common illnesses, determination of referral needs, and education of families for home treatments and health maintenance. Additional economic benefits include reduced travel of university faculty to the site, including lost work (clinical or teaching) time and travel and accommodation costs. A concept of planetary health linking human health, flourishing civilizations and the environment, described in the Lancet commission on planetary health (28), must be considered in our global health work to ensure the health of the environment as well as populations. This is especially true as the people most hurt by climate change are likely to be the communities we are working to help. Using telehealth to provide education allows for reduced carbon emissions related to travel, and if used on a large scale could have a positive effect on the environment. Additionally, this program evaluation started as Zika virus concerns were raised resulting in reduction in travel to endemic locations, including our site where Zika transmission is reported. The ability to deliver education remotely has far reaching benefits from direct costs to planetary health to personal health.

### Limitations

A major limitation to this study was that a specific cost-effectiveness analysis was not performed as the set-up of equipment, bandwidth, licensing of software, and faculty and CHN salaries were all included free-of-charge to our program. While most economic evaluations of telemedicine have failed to show significant cost savings (29), there is a paucity of data overall and especially in the educational (non-direct patient care) usage of telehealth. This is likely due to the fact that equipment, time, and software used are typically shared among projects in an academic setting, and there is no clear tracking of costs spent or saved with the specific telehealth program. Additionally, instructor and learner time commitments were not calculated for our evaluation. While further research exploring cost-effectiveness will be critical to future program expansion and replication, such studies remain a challenge to conduct. This is especially true in the academic setting where the actual costs of shared university resources and faculty time commitments for a specific telehealth program are hard to quantify, making it particularly difficult to estimate the future costs of scale-up, to generalize any findings and to replicate the study results in other non-academic settings.

Another limitation of our study was that the CHNs completed the surveys and tests anonymously, preventing pairing of pre- and posttest scores or the ability to adjust for lecture attendance. This was, however, done to encourage full participation and to remove concerns about job performance, which we feel aided in more candid survey responses. Furthermore, our dataset of surveys and tests was small, as it was designed as a pilot program evaluation, limiting our ability to calculate further correlation or data beyond central tendency. We do not know from our small pilot study whether the knowledge gain by the CHNs translated into better work performance and for what duration of time the knowledge gain persisted. This will be another important area of future exploration.

We are limited in the sustainability of this project which could provide additional quality improvement cycles as well as more detailed data collection, especially as these additional research questions arise. As this program developed in response to an expressed need, our team had several champions for CHN education. This is the main project of a Global Health postdoctoral fellow, who has moved to a different practice location. The program would benefit from an assigned hub of responsibility such as each oncoming fellow, a resident in our global health program or another long-term champion. The project is likely to continue but not necessarily with the same structure or data collection.

### Lessons Learned

Several lectures were followed by informal discussions regarding the overall telehealth experience. These discussions informed the following lectures in an unofficial improvement cycle. Throughout our experience with telehealth for education, we observed that the learners were much more engaged if the didactic portion was limited to 30 min and interspersed with interactive questions supplied by the instructor and real patient cases brought by the CHNs. Engagement was not specifically measured by the instructors but was discussed after lectures among team members. Also, limiting the use of video and audio transmission for those instructors not directly teaching improved the clarity and focus of the lead instructor and allowed the lead instructor to see and hear the learners better. This allowed for reengagement, questions, and breaks as necessary, similar to the way a live teacher can respond to a class. The CHNs reported preference for a larger screen for viewing, especially compared to a small laptop screen as was most commonly used. In the future, it will be important to provide a larger screen and speakers to allow for better video conferencing at the rural LMIC site. Flexibility and patience from both the learners and instructors were also important since some lectures had to be delayed or postponed due to poor connectivity, although this was rare. Anecdotally, the instructors noted that experience with telehealth, especially in understanding the delay in videoconferencing, allowed for better interactions and stronger feelings of connection.

## Conclusion

Our program evaluation shows that telehealth may be an effective and low-cost method of delivering education to frontline health workers, specifically CHNs in rural Guatemala. Post graduate nursing education targeted to current fieldwork was delivered using existing technology systems and easily available resources during work hours with high satisfaction among instructors and learners as well as knowledge improvement among learners from pre- to posttest scores. There are likely to be significant economic benefits to such a telehealth program, including improved fieldwork by CHNs and decreased travel costs by faculty. While larger studies are needed to quantify the duration and benefits of specific knowledge gains and to perform a cost-effectiveness analysis of the program, our initial pilot results are encouraging and show that a telehealth program between an academic university in a high-income country and a rural community health program in a LMIC is both feasible and acceptable. The relationship between the faculty at the US-based Center for Global Health and the frontline health workers at the rural LMIC site in Guatemala is integral to the continued success of this international partnership and community health program, which provides critical health-care access to a rural and impoverished population. The frequent interactions and time and effort given to these educational sessions provide ongoing CHN training in support of the community health program goals and display concern and support for the daily work of the CHNs, contributing to the continuation and longevity of this important partnership.

## Author Contributions

All authors participated in multiple meetings regarding the design of the educational program and the program evaluation. KM was the instructor for all teaching sessions in this program evaluation, with supervision and approval of materials by GD. All authors participated in teaching, primarily MB and ML, prior to this specific program evaluation. LK coordinated educational sessions and analyzed the data with KM. SM, a Guatemala native, assisted with translation and creation of educational materials as well as communication with CHNs. KM did the primary writing, closely working with author GD. All authors reviewed the manuscript and gave final approval.

## Conflict of Interest Statement

The authors declare that the research was conducted in the absence of any commercial or financial relationships that could be construed as a potential conflict of interest.
